# Evaluation of stresses developed in different bracket-cement-enamel systems using finite element analysis with *in vitro* bond strength tests

**DOI:** 10.1186/s40510-014-0033-1

**Published:** 2014-04-16

**Authors:** Shaymaa E Elsaka, Shaza M Hammad, Noha F Ibrahim

**Affiliations:** 1 Department of Dental Biomaterials, Faculty of Dentistry, Mansoura University, Mansoura 35516, Egypt; 2 Department of Orthodontics, Faculty of Dentistry, Mansoura University, Mansoura 35516, Egypt; 3 Department of Production and Mechanical Design, Faculty of Engineering, Mansoura University, Mansoura 35516, Egypt

**Keywords:** Finite element analysis, Orthodontic brackets, Shear, Stresses, Tensile

## Abstract

**Background:**

The purpose of this study was to determine the bond strength of different orthodontic bracket materials (ceramic, stainless steel, and titanium) as well as stresses developed in bracket-cement-enamel systems using finite element (FE) analysis.

**Methods:**

One hundred and thirty-five extracted human caries-free upper central incisors were divided into three groups (*n* = 45/group) according to the type of orthodontic bracket materials (stainless steel, ceramic, and titanium). Each group was further subdivided into three subgroups (*n* = 15/group) according to the bond strength test loading mode (shear short side, shear long side, and tensile). After debonding, the fractured specimen was examined, and the adhesive remnant index (ARI) was determined. FE analysis models analyzed the stress distribution within the cement and enamel. Bond strengths were analyzed using ANOVA and Tukey's test, and the ARI scores were analyzed using chi-square (*χ*^2^) test.

**Results:**

Shear loading at the short side of the bracket resulted in the highest bond strength and lowest maximum principal stress both on cement and enamel compared with the other loading modes (*P* < 0.05). Ceramic brackets presented with higher bond strength and lower maximum principal stress than metallic brackets (*P* < 0.05). There was a significant difference for ARI scores between the type of brackets (*χ*^2^ = 64.852, *P* < 0.001).

**Conclusion:**

The findings suggest that the manner of loading orthodontic brackets and the selection of orthodontic bracket materials affect the bond strength and stresses developed both on cement and enamel.

## Background

The bond strength and the clinical behavior of orthodontic brackets are important to achieve a satisfying orthodontic treatment [1]. Several materials have been used for the production of orthodontic brackets including stainless steel, titanium, plastic, and ceramics. Titanium has been introduced as an alternative material for the production of orthodontic brackets due to its proven biocompatibility, lack of allergenicity, and increased corrosion resistance [2,3]. Ceramic brackets were introduced to orthodontics to meet the increasing demand for more esthetic appliances. In recent years, the number of adults seeking orthodontic treatment has increased, and the need for more esthetic appliances has led manufacturers to design various types of ceramic brackets [4]. However, enamel fractures and cracks have been reported during debonding procedures as ceramic materials are very rigid and brittle materials [5,6].

*In vitro* testing of orthodontic bond strength provides a guide to the selection of bracket-adhesive combinations [7,8]. Measurements of shear and tensile bond strength tests are the most commonly used laboratory assessments to determine the performance of orthodontic bonding systems. Nevertheless, the large distribution of results and the lack of standardization of bond strength testing protocols often prevent confident conclusions from being drawn [8-10].

Finite element (FE) method of stress analysis is a computer-assisted mathematic technique that allows stress levels and distributions to be evaluated in systems with irregular geometry and usually nonhomogeneous physical properties [11]. FE analysis provides an insight into the stress distribution and the strength of bracket-cement-enamel bond. This could result in a better understanding of bracket bond failures and ultimately to prevention of this problem [12]. Consequently, the aim of this study was designed to determine the bond strength of different orthodontic bracket materials (ceramic, stainless steel, and titanium) as well as stresses developed in bracket-cement-enamel systems using FE analysis.

## Methods

### Bonding procedure

One hundred and thirty-five human caries-free upper central incisors, which were extracted due to periodontal disease were collected and stored in phosphate-buffered saline (PBS) (Sigma-Aldrich) at 4°C. The teeth were divided into three groups of 45 teeth for each group of orthodontic bracket materials (stainless steel, ceramic, and titanium) (Table 1). Each group was further subdivided into three subgroups (*n* = 15/group) according to the bond strength test as follows:

*Subgroup 1:* The orthodontic brackets were loaded at the short side during shear bond strength (SBS) test.

*Subgroup 2:* The orthodontic brackets were loaded at the long side during SBS test.

*Subgroup 3:* The orthodontic brackets were loaded using tensile bond strength (TBS) test.

**Table 1:** Materials used in this study with their mechanical properties

**Material**	**Manufacturer**	**Elastic modulus (MPa)**	**Poisson's ratio**
Stainless steel	Victory Series, 3M Unitek, Monrovia, CA, USA. Lot no. 017-663	210,000	0.3
Ceramic	Clarity, 3M Unitek, Monrovia, CA, USA. Lot no. 1564700	380,000	0.29
Titanium	Orthos, Ormco, Glendora, CA, USA. Lot no. 011109087	110,000	0.3
Transbond XT	3M Unitek, Monrovia, CA, USA. Lot no. 6XA/6EB	5,000	0.3
Enamel	-	84,000	0.3

### Specimen preparation

The teeth were embedded in self-cured acrylic resin (Vertex, Vertex-Dental B.V., Zeist, The Netherlands) inside a plastic ring (25 mm in diameter and 20 mm high) to allow standardized and secure placement during testing. Pre-treatment of the bonding area for each type of bracket was carried out according to the manufacturer's instructions. After etching with 37% phosphoric acid gel (Ormco, Orange, CA, USA) for 30 s, the enamel surface was rinsed thoroughly with water and air-dried for 20 s. Transbond XT primer and adhesive (3M Unitek, Monrovia, CA, USA) were applied according to the manufacturer's instructions. Each type of bracket was placed on the tooth and pressed onto the surface. Any excess of the adhesive was removed and the adhesive was cured using a quartz-tungsten halogen curing device (XL2500, 3M ESPE, St. Paul, MN, USA) for 20 s (10 s each for mesial and distal surfaces).

### Shear bond strength and tensile bond strength determination

SBS was determined in two directions. The brackets were loaded at the short and long sides as described by Algera et al. [12,13]. For TBS test, the specimens were attached to the universal testing machine (Model TT-B, Instron Corp., Canton, MA, USA) using a 0.020-in. stainless steel wire bent in a U form and tied with a ligature to the bracket. The free ends of the wire were clamped in the connecting piece of the crosshead, which allowed vertical alignment of the specimen that is required for homogeneous stress distribution during testing [12,14]. The bond strength tests were performed in a universal testing machine at a crosshead speed of 0.5 mm/min. The bond strength in megapascals (MPa) was calculated by dividing the fracture load (*F*) in Newton by the surface area (*A*) in square millimeter. The mean base surface area of the brackets was calculated by measuring the length and width with a digital caliper (Digimatic, Mitutoyo Co., Kawasaki, Japan) and computing the area [15]. After debonding, the fractured specimen was examined, and the adhesive remnant index (ARI) was determined according to Årtun and Bergland [16]:

 0 no adhesive left on the tooth

 1 less than half of the adhesive left on the tooth

 2 more than half of the adhesive left on the tooth

 3 all adhesive left on the tooth, with distinct impression of the bracket mesh.

The ARI scores were used as a more comprehensive means of defining the sites of bond failure between the enamel, resin, and bracket base. The ARI scores were assessed with an optical stereomicroscope (Olympus SZX-ILLB100, Olympus Optical, Tokyo, Japan) with ×20 magnification.

### Statistical analysis

The bond strength mean values were compared using two-way analysis of variance (ANOVA) and a Tukey's multiple comparison test, considering two factors (loading mode and type of bracket) and their interaction. The chi-square (*χ*^2^) test was used to determine if there were any significant differences in the ordinal ARI values. Statistical significance was set at the 0.05 probability level.

### Finite element analysis

**Figure 1:**
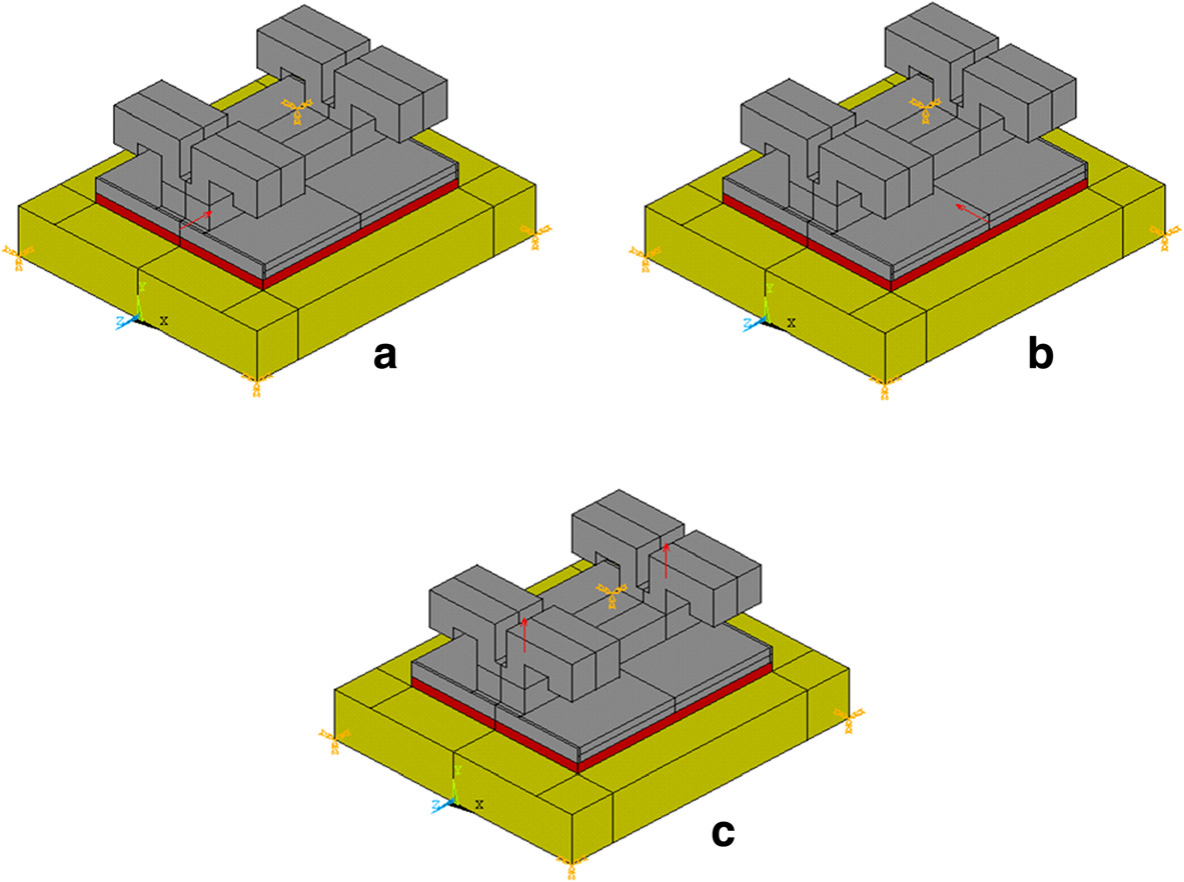
**Representative three-dimensional finite element models of the ceramic bracket-cement-enamel system with different loading modes. (a)** Shear short side, **(b)** shear long side, and **(c)** tensile.

**Figure 2:**
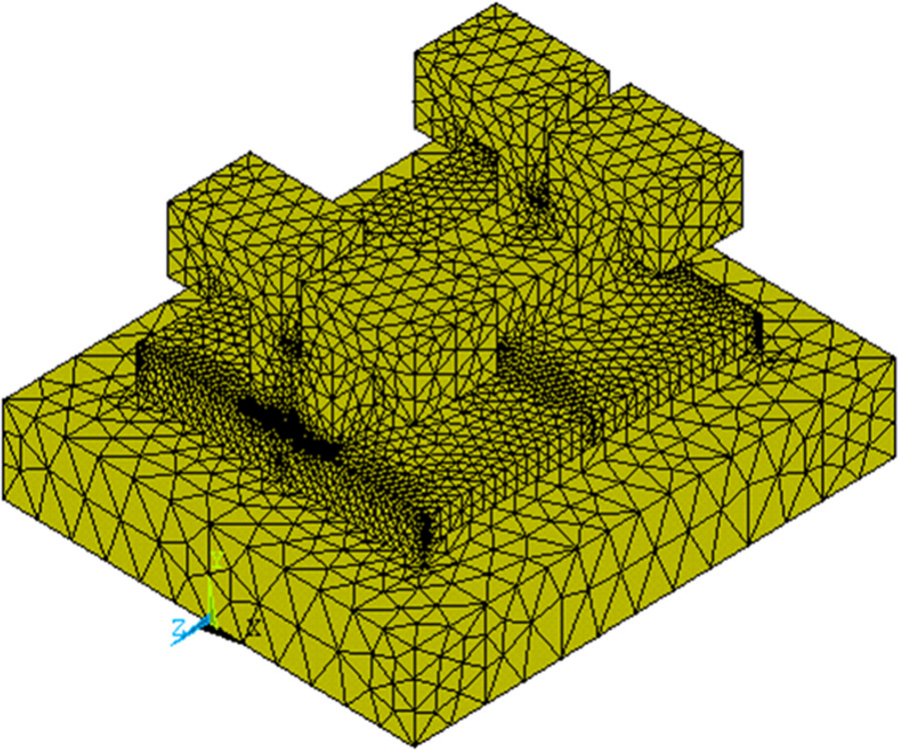
Representative finite element meshes of the ceramic bracket-cement-enamel system.

A three-dimensional simplified FE model with the three loading modes (Figure 1) and the three different bracket materials of the bracket-cement-enamel systems was constructed using ANSYS 10.0 software (ANSYS Inc., Houston, PA, USA). The element type used for this three-dimensional analysis and in constructing the mesh is Solid95. The cement layer was 4.2 mm long, 3.0 mm wide, and 200 μm high. The dimensions of the enamel block were 6.0 mm (length), 5.0 mm (width), and 1.0 mm (height) [12]. The number of elements of stainless steel, ceramic, and titanium bracket models was 68,192, 73,922, and 60,599, respectively, whereas the number of nodes was 99,487, 108,071, and 89,019, respectively. The models were tetrahedral solid elements (Figure 2). The material properties (Table 1) were assumed to be isotropic, homogenous, and linear elastic [17,18]. The nodes at the bottom of the enamel were fixed (no translation or rotation in any direction) [12]. Since this study investigated only the interface between the enamel and adhesive, the enamel was only partially created, and the bottom of the enamel was completely fixed.

## Results

Two-way ANOVA of the bond strength (MPa) testing data (bond tests and bracket materials) revealed that the bond strength was significantly affected by the bond test method and by type of bracket material (*P* < 0.001). There was no significant interaction between the bond test method and type of bracket material (*P* = 0.482) as presented in Table 2. The mean of the three bond strength test method values (MPa) and standard deviations are presented in Table 3. The results of the bond strength showed that the tensile test presented with lower bond strength, whereas the shear bond strength tests showed significantly higher bond strength (*P* < 0.05). Another important finding was that the loading at the short side showed higher bond strength compared with the loading at the long side for shear test methods (*P* < 0.05). For the type of bracket, ceramic bracket showed the highest bond strength followed by stainless steel and titanium brackets in all tested loading modes (Table 3).

**Table 2:** Two-way ANOVA for bond strength test method, bracket material, and interaction terms according to bond strength data

**Source of variation**	**Sum of squares**	***df***	**Mean squares**	***F***	***P*** **value**
Bond strength test method (BT)	845.327	2	422.664	94.436	<0.001
Bracket material (BM)	1,273.965	2	636.983	142.322	<0.001
BT × BM	15.628	4	3.907	0.873	0.482
Total	20,659.891	135			

Statistical significant difference at *P* < 0.05.

**Table 3:** Mean (standard deviation) of the bond strengths (MPa) of different brackets

**Bond strength test mode**	**Type of bracket**		
	**Stainless steel**	**Ceramic**	**Titanium**
Shear strength short side	15.12 (2.34) a A	21.76 (3.43) a B	10.21 (2.10) a C
Shear strength long side	12.15 (2.03) b A	15.25 (2.88) b B	7.69 (1.17) b C
Tensile strength	8.05 (1.24) c A	12.05 (2.26) c B	5.07 (1.11) c C

Mean values represented with common or same uppercase letters (row) are not significantly different according to Tukey's test (*P* > 0.05). Mean values represented with common or same lowercase letters (column) are not significantly different according to Tukey's test (*P* > 0.05).

The ARI scores for the brackets with the three test modes are given in Table 4. The chi-square test showed that significant differences of ARI scores were present between the type of brackets (*χ*^2^ = 64.852, *P* < 0.001). The three test modes did not significantly differ in ARI scores within each type of bracket (*χ*^2^ = 4.831, *P* > 0.05). Regarding the type of bracket, most of the adhesive remained on the enamel for titanium bracket followed by stainless steel bracket, and less adhesive remained on the enamel for ceramic bracket.

**Table 4:** Frequency distribution of adhesive remnant index (ARI) scores

**Group**		**ARI scores**			
		**0**	**1**	**2**	**3**
Stainless steel	Shear short	4	5	6	0
	Shear long	4	6	5	0
	Tensile	0	8	7	0
Ceramic	Shear short	7	6	2	0
	Shear long	6	7	2	0
	Tensile	7	5	3	0
Titanium	Shear short	4	2	3	6
	Shear long	0	4	5	6
	Tensile	0	2	4	9

**Figure 3:**
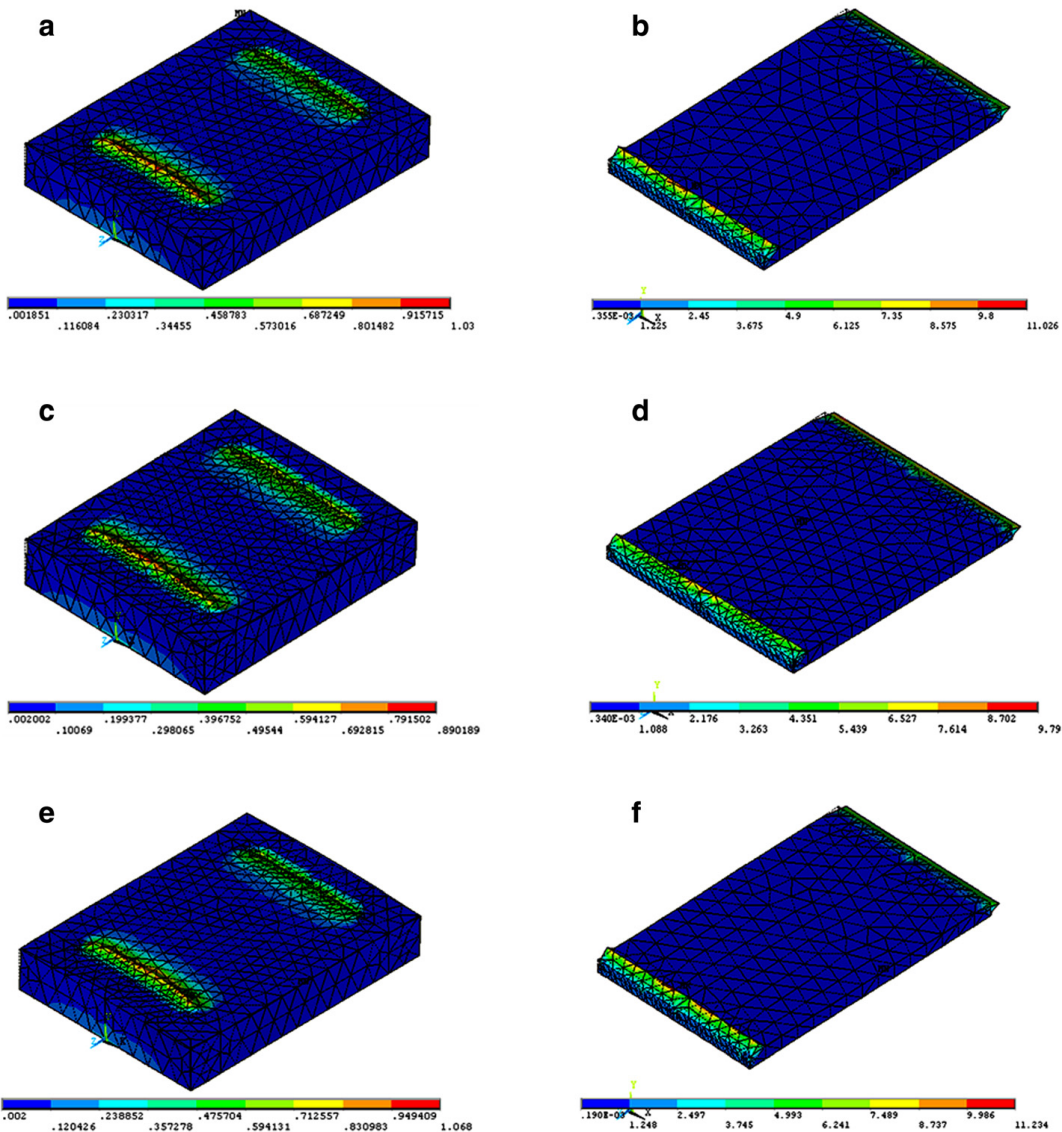
**Maximum principal stress distribution of shear short side bracket loading mode on enamel (left) and cement (right). (a,b)** Stainless steel, **(c,d)** ceramic, and **(e,f)** titanium.

**Figure 4:**
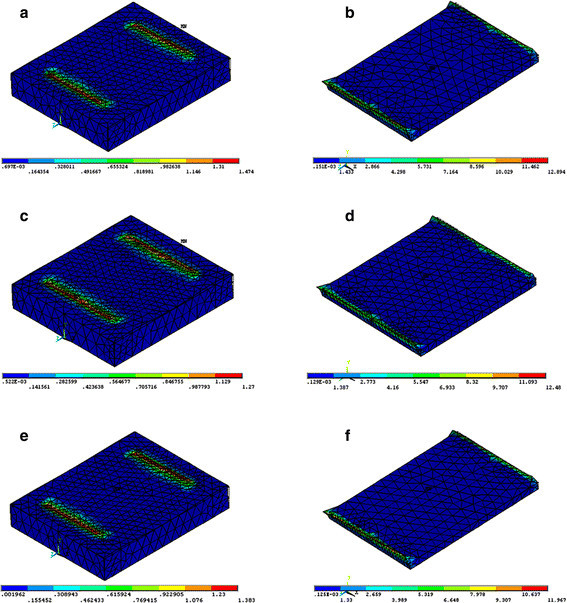
**Maximum principal stress distribution of shear long side bracket loading mode on enamel (left) and cement (right). (a,b)** Stainless steel, **(c,d)** ceramic, and **(e,f)** titanium.

**Figure 5:**
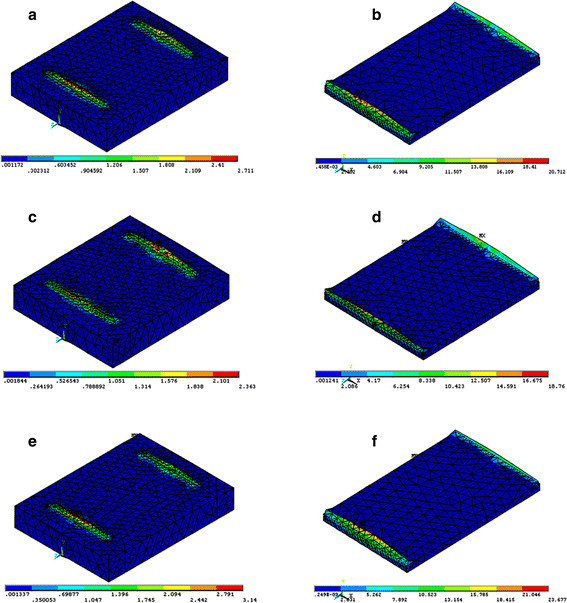
**Maximum principal stress distribution of tensile bracket loading mode on enamel (left) and cement (right). (a,b)** Stainless steel, **(c,d)** ceramic, and **(e,f)** titanium.

The FE model simulation of the sectional views of the cement and enamel for each type of bonded bracket with the different loading modes is presented in Figures 3, 4, 5. The FE analysis indicated that tensile loading resulted in the highest maximum principal stress both on cement and enamel for each type of bracket. Loading the system using shear force on the short side of bracket resulted in the lowest maximum principal stress both on cement and enamel for each type of bracket.

Regarding the type of brackets, the FE analysis showed that the ceramic bracket revealed the lowest maximum principal stress on cement on using the shear short side and tensile loading modes (Figures 3, 4, 5). Ceramic brackets generated the lowest maximum principal stress on enamel compared with the other types of brackets in each loading mode (Figures 3, 4, 5). Titanium bracket showed the highest maximum principal stress on cement and enamel by using the shear short side and tensile loading modes (Figures 3 and 5). Stainless steel bracket presented with the highest maximum principal stresses on cement and enamel by using the shear long side loading mode (Figure 4).

## Discussion

In the present study, the bond strength of different orthodontic bracket materials (ceramic, stainless steel, and titanium) as well as stresses developed in bracket-cement-enamel systems was evaluated using FE analysis. *In vitro* bond strength of the three different types of brackets (ceramic, stainless steel, and titanium) was tested both in shear and in tensile modes. The shear bond strength test was conducted in two modes: at the short and long side of the bracket base. Previous studies reported significant differences in bond strength between shear and tensile tests [12,19], which are in agreement with the results of the present study. In order to understand the peak stress and distribution during loading of the bracket-cement-enamel system, a FE model was performed. To our knowledge, this is the first investigation to evaluate the bond strength and stress distribution of three different types of bracket materials (stainless steel, ceramic, and titanium) using *in vitro* tests and FE models.

Maximum principal stress distribution is the most significant analysis as it is most likely to initiate crack propagation within the brittle cement and enamel [19]. Higher peak stress value is inversely proportional to bond strength [20]. In the present study, a higher maximum principal stress was obtained with the tensile loading mode, and the adhesive layer was prone to fracture as it had lower bond strength than the shear loading mode. In addition, loading at the short side in shear test showed higher bond strength compared with loading at the long side (Table 3). This finding is in agreement with Algera et al. [12]. The maximum principal stress distribution pattern seemed to be correlated with the weak link of the adhesive layer [20].

The FE analysis supported the results of *in vitro* tests for each type of bracket tested in that the higher maximum principal stress resulted in lower bond strength (Figures 3, 4, 5; Table 3). Moreover, regarding the type of bracket material, ceramic brackets revealed the highest bond strength values in all testing modes compared with the other types of brackets, and the FE analysis also supported this finding to a great extent. Ceramic brackets have demonstrated higher bond strengths when compared with metallic brackets in previous studies [21,22]. The bond strength values reported in the present study for ceramic brackets were 12.05, 15.25, and 21.76 MPa (Table 3), which are in agreement with the previously reported values of 10.4, 13.27, and 21.67 MPa [4,23,24]. However, it has been reported that increased bond strength with ceramic brackets resulted in bond failure at the enamel surface, rather than at the bracket-adhesive interface, resulting in more enamel fractures [21,22]. Nevertheless, other studies evaluated the bond strengths of ceramic brackets with different retention mechanisms and found that mechanically retained brackets had adequate bond strength and caused minimal enamel damage [4,25,26]. Our study is in agreement with previous studies [4,25,26] as FE analysis showed that ceramic brackets presented with the lowest maximum principal stresses on enamel in all testing modes, indicating that negligible enamel damage could occur.

In the present study, the bond strength values obtained for the three testing loading modes are above the minimal force levels suggested by Reynolds for a successful clinical debonding (5.9 to 7.8 MPa) [27]. Orthodontic brackets with markedly high bond strength may not be an advantage due to the higher risk of enamel damage during debonding [24]. Retief [28] reported the incidence of enamel fractures in specimens with *in vitro* bond strength values of 9.7 MPa. Even though the enamel can often withstand greater forces as indicated in the debonding force level reported, it is desirable to follow the instructions for debonding as recommended by the manufacturer to avoid enamel damage [24].

ARI scores are influenced by the type of bracket, debonding technique, adhesive type, and the bonding technique used [24,29]. Metallic brackets have a tendency to fail mainly at the bracket-adhesive interface, which leaves the remaining adhesive to be removed from the enamel surface [24,30]. The ARI scores in the present study are in agreement with the findings previously reported for metallic brackets [24,30] as most of the adhesive remained on the enamel for titanium bracket followed by stainless steel bracket. For ceramic bracket, less adhesive remained on the enamel which could probably cause enamel damage; however, in the present study, the ceramic brackets showed the lowest maximum principal stresses on enamel in all testing modes. Consequently, negligible enamel damage could occur, and this finding is in agreement with previous studies [23,25,26]. The use of shear forces to remove orthodontic brackets would result in a reduced risk of enamel fracture when compared to tensile forces (Figures 3, 4, 5). This finding is in agreement with Rossouw and Terblanche [31].

Clinical enhancement of bracket bond strength could be accomplished by altering the bracket design and material which could result in a further homogeneous stress distribution within the cement layer during loading and consequently minimal damage to enamel on debonding orthodontic brackets. Thus, this will provide a guideline for orthodontists for the selection of optimal orthodontic brackets and adhesive for the benefit of the patient at the end. The results of the present study showed that the location of the load, type of bracket material, and accordingly, stress distribution inside the bracket-cement-enamel system are significant parameters in strength testing, selection of type of bracket, and debonding method. Further investigations are needed to evaluate the influence of different bracket base designs and different orthodontic adhesives on the bond strength and stress distribution of orthodontic brackets.

## Conclusions

Based on the results presented and within the limitations of this study, the following conclusions can be made:

1. Ceramic brackets presented with higher bond strength and lower maximum principal stresses on both the cement and enamel compared with metallic brackets.

2. Higher bond strength values were obtained by loading the orthodontic brackets using shear at the short side rather than at the long side.

3. Tensile loading of brackets resulted in lower bond strength with highest maximum principal stresses on both the cement and enamel.

4. Finite element analysis and *in vitro* test results provided a clearer insight of the stress distribution and the strength of bracket-cement-enamel system.

## Competing interests

The authors declare that they have no competing interests.

## Authors' contributions

SE participated in the design of the study, shared in the experimental part, performed the statistical analysis, and drafted the manuscript. SH participated in the design of the study and shared in the experimental part. NF participated in the design of the study and shared in the experimental part (performed the finite element analysis). All authors read and approved the final manuscript.
